# Molecular mechanism of sequence-dependent stability of RecA filament

**DOI:** 10.1093/nar/gkt570

**Published:** 2013-06-26

**Authors:** Sung Hyun Kim, Chirlmin Joo, Taekjip Ha, Doseok Kim

**Affiliations:** ^1^Department of Physics and Interdisciplinary Program of Integrated Biotechnology, Sogang University, Seoul 121-742, Korea, ^2^Kavli Institute of NanoScience, Department of BioNanoScience, Delft University of Technology, 2628 CJ, Delft, The Netherlands, ^3^Department of Physics and Center for the Physics of Living Cells, Institute for Genomic Biology and Center for Biophysics and Computational Biology, University of Illinois at Urbana-Champaign, Urbana, IL 61801, USA and ^4^Howard Hughes Medical Institute, Urbana, IL 61801, USA

## Abstract

RecA is a DNA-dependent ATPase and mediates homologous recombination by first forming a filament on a single-stranded (ss) DNA. RecA binds preferentially to TGG repeat sequence, which resembles the recombination hot spot Chi (5′-GCTGGTGG-3′) and is the most frequent pattern (GTG) of the codon usage in *Escherichia coli*. Because of the highly dynamic nature of RecA filament formation, which consists of filament nucleation, growth and shrinkage, we need experimental approaches that can resolve each of these processes separately to gain detailed insights into the molecular mechanism of sequence preference. By using a single-molecule fluorescence assay, we examined the effect of sequence on individual stages of nucleation, monomer binding and dissociation. We found that RecA does not recognize the Chi sequence as a nucleation site. In contrast, we observed that it is the reduced monomer dissociation that mainly determines the high filament stability on TGG repeats. This sequence dependence of monomer dissociation is well-correlated with that of ATP hydrolysis, suggesting that DNA sequence dictates filament stability through modulation of ATP hydrolysis.

## INTRODUCTION

RecA is a DNA-dependent ATPase and mediates homologous recombination by forming a helically structured filament on a single-stranded (ss) DNA (1). In a RecA nucleoprotein filament, six monomers make one helical turn while each monomer occupies three nucleotides (2,3). Filament formation is initiated by the nucleation of a group of RecA monomers (more than five) with an ATP molecule as a cofactor for each monomer (4,5). The nucleated short filament is unstable and quickly disassembles from DNA unless the filament is further extended by adding monomers at the ends.

RecA filament grows rapidly toward the 3′ end of the bound ssDNA and shrinks from the 5′ end (6). A monomer may dissociate from the ends of a stable filament on ATP hydrolysis (4,7–11). The dissociation rates are identical at both ends, but the monomer addition rate is 10-fold higher for the 3′ end (4). Thus, it is the difference between the binding rate and the dissociation rate that determines filament growth and shrinkage at each end. The 3′ end of the filament is relatively stable owing to the higher binding rate than the dissociation rate, whereas the 5′ end is less stable at the physiological RecA concentration owing to the comparable binding and dissociation rates.

Early studies found that the DNA-binding affinity and ATPase activity of RecA depend on DNA sequence (12–14). It was suggested that the breaking of secondary structures and base stacking of purine-rich sequences are the major obstacles against RecA polymerization and therefore are responsible for the sequence dependence (15). However, secondary structures and base stacking interactions can be effectively diminished by single-stranded binding proteins (16). Also, this model cannot explain the sequence dependence of ATPase activity. Therefore, there may be other factors that determine the sequence preference such as changes in intrinsic binding affinity or in enzymatic activity via direct interaction between DNA bases and RecA proteins.

Previous studies carried out in-depth characterization of the sequence preference using repeats of three nucleotides. *In vitro* selection found preferential binding of RecA to TGG repeats (or TG-rich sequence) (17,18). A similar preference was reported for the coprotease activity of RecA (19). The authors in these studies used ‘TGG repeats’ and ‘TG-rich sequence’ interchangeably. We believe that a repeat of tri-nucleotide is more relevant term because RecA generates groups of three nucleotides within a filament (20), and the tri-nucleotide repeat is likely to be a DNA manipulation unit of a filament.

Because of the striking resemblance of the TGG repeats and the recombination hotspot Chi (5′-GCTGGTGG-3′), it was suggested that the Chi sequence may play a role for RecA-mediated strand exchange (18,19,21). It still needs to be confirmed whether this resemblance is just a coincidence or the Chi sequence has any specific functional role such as being a preferred nucleation site. Additionally, because GTG, a permutation of TGG, is the most frequent pattern of the codon usage in *Escherichia coli* (22,23), RecA may have evolved in such a way that it preferentially binds to the coding region of genome.

RecA filament can undergo a complex dynamic process that consists of nucleation, ATP hydrolysis and filament growth and shrinkage. Challenges in studying the sequence-dependent RecA filament dynamics come from this complexity even though various experimental approaches have been used in the past decades (12–15,17–19,21). It is necessary to have an appropriate experimental tool to resolve each process separately for a deeper understanding of the sequence-dependent RecA filament dynamics. In this report, we applied the method of single-molecule fluorescence resonance energy transfer (FRET) (24), which has been used to study various protein dynamics on single-stranded DNA (25), in particular, the RecA filament dynamics (4,26), RecA-mediated strand exchange (27) and sliding of a RecA filament on DNA during homology search (28). We directly observed nucleation events on different sequences in real-time exclusive of the other filament dynamics. We have also separately measured the monomer binding and dissociation events that occur after nucleation. We found that the nucleation frequency is inversely correlated with the disassembly rate of the nucleated cluster in a sequence-dependent manner. However, we did not observe any notable change in the nucleation process with the Chi sequence. Monomer dissociation at a filament end was the slowest with TGG repeats, whereas monomer binding rates showed only weak sequence dependence. The sequence dependence of monomer dissociation at 5′ end is well correlated with ATP hydrolysis rates, implying that the sequence-dependent ATPase activity is the main factor that determines the stability of a RecA filament.

## MATERIALS AND METHODS

### Reaction condition

RecA was purchased from New England Biolabs and used without further purification. Reaction was performed with a solution consisting of 1 μM RecA, 10 mM Mg(OAc)_2_, 100 mM NaOAc and 10 mM Tris–OAc at pH 7.5 with 1 mM ATP (Sigma) at room temperature. The reaction buffer also contained 2 mg/ml 6-hydroxy-2,5,7,8-tetramethylchroman-2-carboxylic acid (Sigma), 1 mg/ml glucose oxidase (Sigma), 0.8 % (w/v) dextrose (Sigma) and 0.04 mg/ml catalase (Sigma) to enhance the photo stability of the dyes.

### DNA

Single-stranded DNA molecules were purchased from IDTDNA. A DNA with the sequence of 5′-Cy5-GCC TCG CTG CCG TCG CCA –biotin-3′ was labeled with Cy5 and biotin during the DNA synthesis. For the nucleation measurement, the Cy5 labeled DNA was hybridized with the sequence of 5′-TGG CGA CGG CAG CGA GGC-(XYZ)_7_-Cy3-3′, where XYZ indicates the repeated tri-nucleotide sequences of TTT, TTG, TCA or CCA. We also prepared the complementary sequence containing one copy of the Chi sequence (5′-TGG CGA CGG CAG CGA GGC-T_7_-GCTGGTGG-T_6_-Cy3-3′) and two copies of the Chi sequence (5′-TGG CGA CGG CAG CGA GGC TTG CTG GTG GTT TTG CTG GTG GT TT-Cy3-3′). For the monomer dynamics experiments, we prepared complementary sequences with long 3′ single-strand tail: 5′-TGG CGA CGG CAG CGA GGC-Z(XYZ)_3_-T*-(dT)_49_-3′, where the star denotes the amine-labeled base for the Cy3 labeling. Here, XYZ combinations are TTT, TGG, TTG, TCA and CCA. Cy3 was purchased from GE healthcare, and the labeling was performed following the protocol that the company provided. The hybridized DNA was immobilized via Neutravidin-biotin linker on the polyethylene glycol-coated quartz surface to avoid non-specific adsorption of RecA. For the ATPase rate measurement, we used 48 nt tri-nucleotide repeats: (TGG)_16_, (TTG)_16_, (TTT)_16_, (TCA)_16_ and (CCA)_16_.

### Single-molecule FRET assay

Experimental details on single-molecule FRET assay are described in the previous work (4). The dye-labeled duplex DNA immobilized on quartz slide surface was illuminated by 532 nm laser (CrystaLaser) via total internal reflection to minimize background noise. Fluorescence signals from the donor and acceptor were collected with an objective lens (Olympus, NA 1.2 water immersion) and separated spectrally with a dichroic mirror before being imaged on an electron-multiplying charge coupled device. Fluorescence intensity time traces were extracted out from a movie taken, and FRET efficiency was calculated with home-built software. Most likely, FRET time trajectories were estimated with hidden Markov method (29). FRET histograms were built from thousands of single molecules, of which the FRET values were determined by averaging the first 10 data points from the traces. All measurements were carried out at the room temperature.

### ATPase rate measurement

ATPase activity was measured with EnzChek kit (E-6646, Invitrogen) and a dual-beam UV-spectrometer (UV-2450, Shimadzu). In all, 100 nM of unlabeled 48mer ssDNA was mixed with 1 uM RecA and 1 mM ATP in the identical chemical condition as single-molecule measurements at the room temperature. The rates were determined by the slope in the linear region of the time-dependent absorption changes at 360 nm.

## RESULTS

### Direct measurement of nucleation process

RecA filament formation is initiated by nucleation of several RecA monomers on a DNA strand. Afterwards, the filament may grow or shrink by monomer binding and dissociation at the ends. Because the sequence of a substrate DNA may affect each of these processes in different ways, we need to investigate these effects separately. To this end, we used a single molecule fluorescence resonance energy transfer (smFRET) assay to examine each process independently (24).

**Figure 1.**
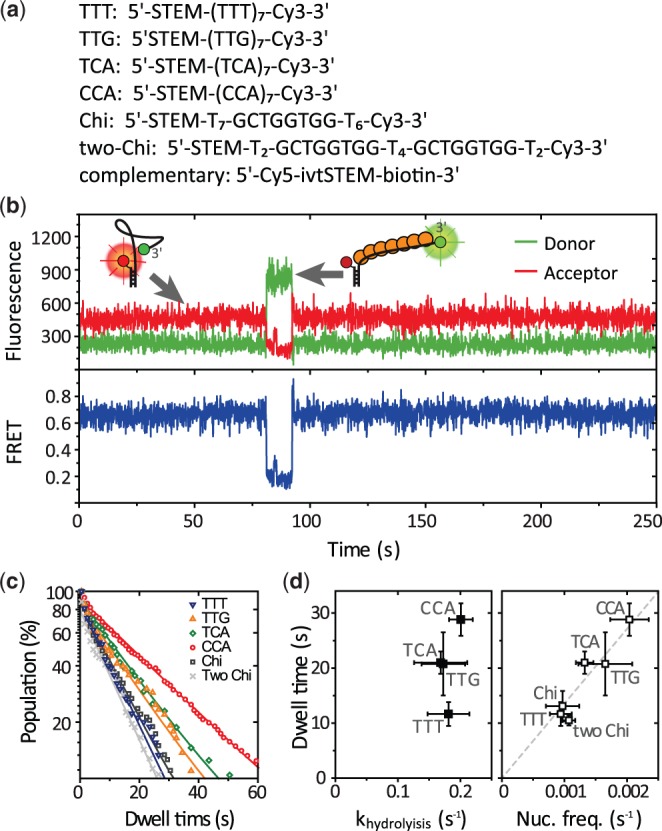
Sequence-dependent RecA nucleation process. (**a**) DNA sequences and dye-labeling positions. STEM indicates 18 nt long random sequences for hybridization with its complementary sequence (ivtSTEM) labeled with a Cy5 and biotin for surface immobilization. (**b**) An example FRET time trace from d(TCA)_7_. Inset: a schematic of RecA filament nucleation. (**c**) Dwell time histograms from six different sequences. They were fitted with a single exponential decay curve. More than 100 nucleation events were used for each plot. (**d**) A correlation plot of the dwell time versus the ATP hydrolysis rate (left panel); and the dwell time versus the nucleation frequency (right panel). Error bars are standard deviations from three independent data sets.

First, we examined the effect of DNA sequence on the nucleation process. We designed a partial duplex DNA with 21 nt long 3′ ssDNA tail composed of the seven repeats of tri-nucleotides (TCA, CCA, TTT and TTG). This length is sufficient for filament nucleation but not enough for further growth (Figure 1a). We also prepared a duplex DNA with its ssDNA tail containing the recombination hot spot Chi (5′-GCTGGTGG) to check the effect of the Chi on the RecA nucleation process. We could not perform the measurement with (TGG)_7_ because it showed a complex FRET distribution even as bare ssDNA, which appears to be due to its G-rich context. Fluorescent donor (Cy3) was attached at the end of the ssDNA tail, and acceptor (Cy5) was placed at the junction of the tail and the duplex region. Because ssDNA is highly flexible and its conformations are averaged within our time resolution (typically 0.1 s) (30), the time-averaged inter-dye distance increases on RecA binding that stretches its bound region. FRET changes, therefore, reflect RecA–ssDNA interaction at the ssDNA tail.

We immobilized the DNA constructs on a polyethylene glycol-coated quartz surface and measured fluorescence signals by using a total internal reflection microscope. Shown in Figure 1b are typical single molecule fluorescence (top) and FRET (bottom) time traces. FRET efficiency of the bare 21 nt ssDNA was ∼0.7 and suddenly dropped to ∼0.2 on nucleation of a RecA filament and returned to ∼0.7 when on disassembly (Figure 1b). The transitions were observed as single steps in our time resolution (0.1 s), implying that the nucleation of a filament and its disassembly occur as a multimer, likely ∼6 monomers (4). To quantitatively analyze the stability of the nucleated short filament, we collected the dwell times of the bound state (Figure 1c). The average dwell time varied among different DNA sequences with the highest stability observed from the CCA repeats and the lowest from the TTT repeats. The average dwell time of the nucleated filament showed no correlation with the ATP hydrolysis rate measured with the same set of tri-nucleotide repeats (Figure 1d, left).

We calculated the frequencies of nucleation events and plotted them against the average lifetimes of the nucleated filament. The data show that the sequences that gave higher frequencies of nucleation also made the disassembly of the nucleation cluster slower (Figure 1d, right), suggesting that the transition state between the bound and unbound states share more similarities to the bound state than the unbound state (see ‘Discussion’ section for detail).

We note that TG-rich sequence, d(TTG)_7_, did not show any particularly enhanced nucleation frequency or elongated nucleation dwell time as compared with the other sequences (Figure 1c and d). Similarly, the nucleation frequency and the dwell time are not affected by the presence of Chi sequence (Figure 1c and d, ‘Chi’). Presence of two copies of Chi sequence did not alter the kinetics either (Figure 1c and d right, ‘two Chi’). These results show that the previously reported sequence preference on TGG repeats (or TG rich sequence) is not related to the nucleation process.

### Monomer binding and dissociation observed with single monomer resolution

**Figure 2.**
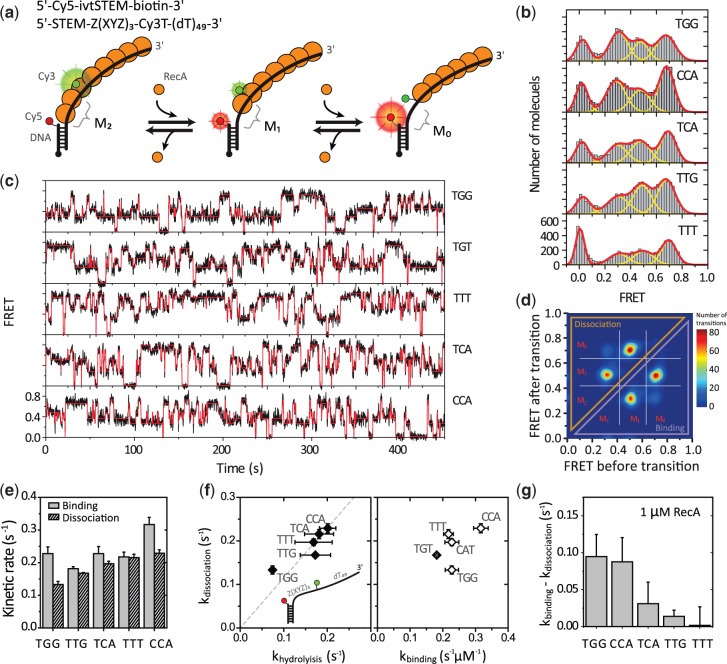
Sequence-dependent filament dynamics at the 5′-disassembly end. (**a**) Top: DNA sequences and dye-labeling positions. Bottom: Illustrated is a schematic of RecA monomer binding and dissociation at the 5′ end of a filament. M2: two monomers between a pair of dyes. M1: one monomer. M0: no monomer. (**b**) smFRET histograms with five different DNA sequences. The three distinct FRET populations were fitted with Gaussian peaks (yellow lines for individual peaks and a red line for the sum of the peaks) with an additional peak at zero from donor-only molecules. (**c**) Typical FRET time traces (black lines) obtained with different sequence combinations. The transition points of monomer binding and dissociation were determined with HMM (red lines). (**d**) An example TDP of 7560 transitions from 94 molecules with TTT. (**e**) The monomer binding and dissociation rates obtained with the HMM. (**f**) A correlation plot of the dissociation rate versus the ATPase rate (left) and the dissociation rate versus the binding rate for each sequence (right). The broken line is the linear fit of the data. Error bars are standard deviations from three data sets. (**g**) The apparent shrinkage rates of a filament at the 5′ end. It was obtained by subtracting the dissociation rate from the binding rate in (e) (*k_shrinkage_ = k_on_ × [RecA] − k_off_*).

Next, to examine the dynamics at the filament end at which monomer binding and dissociation occur, we prepared a partial duplex DNA with a long 3′ ssDNA tail (>60 nt) to form a stable filament such that only the monomers at the ends can dissociate (Figure 2a). We then placed a dye pair at the 5′ end of the single-strand tail; that is, the acceptor was attached at the junction between ss- and dsDNA, and the donor was attached at the tail with 10-nt separation from the acceptor. As the 3′ side of the filament is much more stable at the RecA concentration used in this study (1 μM) owing to the faster binding rate at the 3′ end, it is only at the 5′-disassembly end where monomer binding and dissociation occur repeatedly (4). We changed the sequence between the two fluorophores with different repeats of tri-nucleotides; TGG (corresponding to GTG and GGT in permutation), TTG (TGT, GTT in permutation), TTT, CCA (CAC, ACC in permutation) and TCA (CAT, CTC in permutation). To minimize the effect of potential secondary structures, we placed the repeats only between the two fluorophores while keeping the rest of the tail as poly-thymine.

The smFRET histograms showed broad distribution for all the sequence combinations we tested (Figure 2b). Three peaks emerged at 0.3, 0.5 and 0.7, each of which corresponds to 2, 1 and 0 monomer occupancy between the dye pair, respectively. The peak that appeared at 0 is from donor-only molecules. For a more quantitative analysis, we recorded individual monomer binding and dissociation events in real time. Shown in Figure 2c are typical smFRET time traces from single RecA filaments. Sudden changes in FRET efficiency in the time trajectories correspond to binding and dissociation of a single monomer (Δ*E*∼0.2 for each transition). With hidden Markov modeling (HMM) analysis, we determined the most likely idealized trajectory that represents transitions between discrete states (red solid line) (29).

We compiled all identified transitions on a transition density plot (TDP) to classify the transitions. As an example, a TDP plot with TTT repeats is shown in Figure 2d. In the four peaks shown, two represent binding events and the other two represent dissociation. As we reported previously (4), we observed binding of only two monomers, not the maximum three monomers expected for 10 nt, possibly because of steric hindrance by the ss-dsDNA junction (4). We designate M_0_, M_1_ and M_2_ (*E*∼0.7, 0.5 and 0.3) as zero, one and two monomer-bound states of the 10 nt segment, respectively. Average rates of transitions between these states were calculated from the values defined by HMM. A state of *E*∼0 arises from acceptor photo-inactivation and was excluded in calculating the kinetic rates.

### Monomer dissociation is slowest with TGG repeats

The monomer binding and dissociation rates from the 5′ disassembly end showed remarkable sequence dependence (Figure 2e). Notably, the TGG repeat showed the slowest dissociation as compared with the others. This result is consistent with previous reports that stable filament formation is observed with TGG repeats (18,19). In addition, the dissociation rate we determined is well correlated with the ATPase rates, suggesting that ATP hydrolysis is a rate-determining step in RecA monomer dissociation (Figure 2f, left; *R^2^* = 0.82). This result implies that the sequence dependence of monomer dissociation originates from the difference in ATPase activity for different sequences. The binding rate also varied with different substrate sequences but was less correlated with the dissociation rate (Figure 2f, right; *R^2^* = 0.45).

## DISCUSSION

We explored the sequence-dependent RecA filament dynamics by separately measuring nucleation and the dynamics at the filament ends using a single-molecule fluorescence technique. The nucleation frequency is strongly correlated with the lifetime of the nucleated filament. We did not observe any enhancement of the filament nucleation with either TG-rich or Chi containing DNA. At the filament end, the dissociation rate showed a strong correlation with the ATP hydrolysis in a sequence-dependent manner. Interestingly, we observed the slowest monomer dissociation from the TGG repeats. Later in the text, we describe the mechanistic implications of our findings *in vitro* and *in vivo*.

## Kinetic models for nucleation and monomer dynamics

The frequencies of nucleation are correlated with the lifetimes of the nucleated filament when the sequence of substrate DNA is varied (Figure 1c). This result can be explained with a simple binding and dissociation model of two chemical species [*A* + *B* ↔ *AB*], which does not involve any enzymatic activity of a RecA protein. In this model, the sequence of a substrate DNA alters the thermodynamic energy of the bound state, resulting in the change of both binding and dissociation kinetics. For example, when we change the sequence from CCA to TTT, the transition rate from the bound state to the unbound state increased and the transition rate from the unbound state to the bound state increased. Therefore, it is likely that the transition state between the bound and unbound states is more similar in its properties to the bound state. The dwell times of the nucleated filament are not correlated with ATP hydrolysis rates (Figure 1d, left), implying that the ATP hydrolysis is not involved in the rate-determining step in this nucleated filament disassembly even though ATP hydrolysis is a prerequisite to the disassembly (26).

We showed that the monomer binding and the dissociation processes at a filament end also have their own sequence dependence. The dissociation rate was found to be correlated with the ATP hydrolysis rate (Figure 2e and f). On the other hand, the binding and the dissociation were not correlated with each other; thereby, these two processes are not a reverse process of each other. Unlike the nucleation process, the monomer dynamics is explained by the enzyme (E) catalyzed reaction model [*E+S→ ES → E+P*] in which monomer binding [*E+S → ES*] is governed by the intrinsic affinity of a RecA monomer to the filament end, whereas monomer dissociation [*ES → E+P*] depends on the enzymatic activity (ATP hydrolysis of the end monomer). It is natural to see that these two independent processes, the binding and the dissociation, have different sequence preference.

## Filament growth and shrinkage

As a measure of filament stability, we calculated the rate of filament growth based on the difference between the binding and dissociation rates (*k_growth_ = k_on_ × [RecA] −k_off_*, Figure 2e). The monomer binding rate depends on the RecA concentration, but the dissociation rate does not. At the critical concentration of RecA, at which the binding and the dissociation rates are the same, both binding and dissociation terms contribute equally, and therefore the concentration of RecA becomes the most important parameter that determines the filament stability. If the RecA concentration is far below the critical concentration, the binding rate becomes negligible, whereas the dissociation remains as a dominant term, which will solely determine filament stability. For example, the filament growth rates on the TGG repeat and the CCA repeat were comparable when the critical concentration of RecA (∼1 µM) was provided (Figure 2g). But, at a low RecA concentration, the dissociation term becomes dominant in the growth rate, and thus TGG becomes the most stable sequence and CCA becomes the most unstable (Figure 2e). This result emphasize that the sequence dependence of a RecA filament is highly environment dependent, and thus the experimental condition must be explicitly specified and carefully controlled.

## *In vivo* implications of sequence dependence

RecA-binding rate at the 3′ end is almost 10-fold higher than the disassembly rate of a short nucleated filament in the physiological concentration of RecA (∼ 1 μM) (4). Thus, nucleation events would readily lead to extension toward the 3′ end and formation of a stable long RecA filament. Thus, the modest sequence dependence of disassembly rate of a short nucleated filament we observed may not have much effect *in vivo*. Even though the nucleation process is the rate-limiting step of the RecA filament formation in *in vitro* experiments, nucleation *in vivo* is facilitated by RecA-loading machineries such as RecBCD and RecFOR (31). Therefore, it is not surprising that we did not observe any enhancement on the nucleation process in the presence of Chi sequence (Figure 2c).

In contrast, kinetics of monomer binding and dissociation at the 5′ end is directly related to the filament disassembly and determines the overall filament stability. As we pointed out earlier in the text, filament dynamics is characterized by the competition between monomer binding and dissociation processes. At low RecA concentrations, monomer dissociation would dominantly occur from the 5′ end, and sequence dependence of dissociation will be the most important parameter regarding the filament stability. Thus, the reduced dissociation rate solely explains stable filament formation on TGG repeats.

We showed that RecA does not recognize Chi sequence as a preferred nucleation site. However, the pattern of TGG repeats is found across thousands of bases near Chi sites, and it is considered that this TG-rich region makes the filament stable *in vivo* (32). Along the same line, this preferred sequence of TGG (GTG and GGT in permutation) is the most frequent pattern of the codon usage (GTG) in *E. coli* gene (22,23). Together with these interesting facts, our results suggest the following biological implication of the sequence preference of RecA: RecA might have evolved to form a more stable filament around a coding region by reducing the ATP hydrolysis (thus reducing monomer dissociation). This will promote efficient homologous search and strand exchange reaction over the coding region.

## CONCLUSION

By observing binding and dissociation of individual monomers at a filament end, we showed that the stability of a RecA filament on a TGG repeat sequence originates from reduced monomer dissociation, which is coupled with reduced ATPase activity. Our study demonstrates that the molecular mechanism of RecA filament stability is best understood when separate observations are made for the nucleation process and the monomer binding and dissociation processes.

## FUNDING

Korean Government Ministry of Education and Science Technology (MEST) [2011-0017435], Sogang University Research [SRF-20124003 to D.K.]; US National Science Foundation [PHY-0646550, PHY-0822613 to T.H.]; US National Institutes of Health [GM065367 to T.H.]; European Research Council under the European Union’s Seventh Framework Programme [FP7/2007-2013] / ERC grant agreement n° [309509] (to C.J.). Funding for open access charge: Korean Government (MEST) [2011-0017435].

*Conflict of interest statement.* None declared.
